# Supplementary material for: Evaluation of systems reform in public hospitals, Victoria, Australia, to improve access to antenatal care for women of refugee background: An interrupted time series design
Source: PLoS Med. 2020 Jul 10;17(7):e1003089. doi: 10.1371/journal.pmed.1003089 (PMC7351141; doi:10.1371/journal.pmed.1003089)
Supplement: S1 Table — (DOCX) [file pmed.1003089.s003.docx]

**S1 Table: Country of birth for women of refugee background by year for Hospital Networks X and Y**

| **Country of Birth** | **Hospital Network X** | | | | **Hospital Network Y** | | | |
| --- | --- | --- | --- | --- | --- | --- | --- | --- |
|  | **2014** | **2015** | **2016** | **Total** | **2014** | **2015** | **2016** | **Total** |
|  | **n (%)** | **n (%)** | **n (%)** | **n (100%)** | **n (%)** | **n (%)** | **n (%)** | **n (100%)** |
| Afghanistan | 435 (28.8) | 493(32.6) | 583(38.6) | 1511 | 15(28.3) | 10(18.9) | 28(52.8) | 53 |
| Sudan | 124(29.2) | 154(36.3) | 146(34.4) | 424 | 160(31.6) | 174(34.3) | 173(34.1) | 507 |
| Burma (Myanmar) | 49(29.0) | 64(37.9) | 56(33.1) | 169 | 100(34.8) | 85(29.6) | 102(35.5) | 287 |
| Iran | 53(36.1) | 48(32.7) | 46(31.3) | 147 | 23(38.3) | 25(41.7) | 12(20.0) | 60 |
| Iraq | 31(31.0) | 28(28.0) | 41(41.0) | 100 | 24(28.6) | 25(29.8) | 35(41.7) | 84 |
| Sri Lanka (Tamil only) | 48(35.8) | 38(28.4) | 48(35.8) | 134 | 14(41.2) | 9(26.5) | 11(32.4) | 34 |
| Somalia | 0 | 0 | 0 | 0 | 33(40.7) | 22(27.2) | 26(32.1) | 81 |
| Ethiopia | 36(27.1) | 43(32.3) | 54(40.6) | 133 | 0 | 0 | 0 | 0 |
| Congo | 0 | 0 | 0 | 0 | 17(29.3) | 22(37.9) | 19(32.8) | 58 |
| Eritrea | 3(27.3) | 4(36.4) | 4(36.4) | 11 | 14(35.0) | 13(32.5) | 13(32.5) | 40 |
| Liberia | 6(17.1) | 15(42.9) | 14(40.0) | 35 | 13(32.5) | 17(42.5) | 10(25.0) | 40 |
| Other | 25(32.9) | 24(31.6) | 27(35.5) | 76 | 58(34.1) | 47(27.6) | 65(38.2) | 170 |
