# Supplementary material for: Evaluation of systems reform in public hospitals, Victoria, Australia, to improve access to antenatal care for women of refugee background: An interrupted time series design
Source: PLoS Med. 2020 Jul 10;17(7):e1003089. doi: 10.1371/journal.pmed.1003089 (PMC7351141; doi:10.1371/journal.pmed.1003089)
Supplement: S2 Table — (DOCX) [file pmed.1003089.s004.docx]

**S2 Table: Mean antenatal visits by time period comparing Australian-born women and women of refugee background (January 2014 to December 2016)**

| **Hospital Network X** | | | | | | | | | | | |
| --- | --- | --- | --- | --- | --- | --- | --- | --- | --- | --- | --- |
|  | **Australian-born women** | | | | | | **Women of refugee background** | | | | |
|  | **n (%)** | | **M (SD)** | **Median** | **IQR** | **Range** | **n (%)** | **M (SD)** | **Median** | **IQR** | **Range** |
| 1. Baseline 1 | 1,650 (16.1) | | 7.09 (4.1) | 8 | 4-10 | 0-21 | 402 (14.7) | 7.29 (3.8) | 8 | 5-10 | 0-24 |
| 2. Baseline 2 | 1,707 (16.6) | | 8.41 (3.4) | 9 | 6-10 | 0-23 | 408 (14.9) | 8.48 (3.3) | 9 | 7-10 | 0-20 |
| 3. Intervention 1 | 1,657 (16.1) | | 8.62 (3.8) | 9 | 6-11 | 0-28 | 458 (16.7) | 8.39 (3.3) | 8.5 | 6-10 | 1-20 |
| 4. Intervention 2 | 1,777 (17.3) | | 9 (3.6) | 9 | 7-11 | 0-30 | 453 (16.5) | 8.73 (3.2) | 9 | 7-10 | 1-26 |
| 5. Intervention 3 | 1,704 (16.6) | | 9.06 (3.6) | 9 | 7-11 | 0-30 | 514 (18.8) | 8.73 (3) | 9 | 7-10 | 1-21 |
| 6. Intervention 4 | 1,782 (17.3) | | 9.01 (3.3) | 9 | 7-11 | 0-30 | 505 (18.4) | 8.9 (3.2) | 9 | 7-10 | 0-25 |
|  |  | |  |  |  |  |  |  |  |  |  |
| **Total** | **10,277 (100.0)** | | **8.55 (3.7)** | **9** | **7-11** | **0-30** | **2,740 (100.0)** | **8.46 (3.3)** | **9** | **7-10** | **0-26** |
|  | | | | | | | | | | | |
| **Hospital Network Y** | | | | | | | | | | | |
|  | | **Australian-born women** | | | | | **Women of refugee background** | | | | |
|  | | **n (%)** | **M (SD)** | **Median** | **IQR** | **Range** | **n (%)** | **M (SD)** | **Median** | **IQR** | **Range** |
|  | |  |  |  |  |  |  |  |  |  |  |
| 1. Baseline 1 | | na | na | na | na | na | na | na | na | na | na |
| 2. Baseline 2 | | 791 (16.1) | 7.87 (4.3) | 8 | 5-10 | 0-27 | 161 (15.0) | 7.92 (4.1) | 8 | 5-10 | 0-30 |
| 3. Intervention 1 | | 878 (17.8) | 7.53 (4.8) | 8 | 4-11 | 0-25 | 179 (16.7) | 7.87 (4.2) | 8 | 5-11 | 0-19 |
| 4. Intervention 2 | | 1,091 (22.1) | 8.23 (4.9) | 8 | 5-11 | 0-30 | 236 (22.1) | 8.22 (3.6) | 8 | 6-10 | 0-24 |
| 5. Intervention 3 | | 1,049 (21.3) | 8.1 (4.3) | 8 | 6-10 | 0-30 | 273 (25.5) | 7.2 (3.5) | 7 | 5-9 | 0-21 |
| 6. Intervention 4 | | 1,116 (22.7) | 8.02 (4.2) | 8 | 6-10 | 0-30 | 221 (20.7) | 7.65 (3.7) | 8 | 6-9 | 0-28 |
|  | |  |  |  |  |  |  |  |  |  |  |
| **Total** | | **4,925 (100.0)** | **7.95 (4.5)** | **8** | **5-10** | **0-30** | **1,070 (100.0)** | **7.74 (3.8)** | **8** | **6-10** | **0-30** |
