# Supplementary material for: Evaluation of systems reform in public hospitals, Victoria, Australia, to improve access to antenatal care for women of refugee background: An interrupted time series design
Source: PLoS Med. 2020 Jul 10;17(7):e1003089. doi: 10.1371/journal.pmed.1003089 (PMC7351141; doi:10.1371/journal.pmed.1003089)
Supplement: S3 Table — (DOCX) [file pmed.1003089.s005.docx]

**S3 Table: Number antenatal visits and gestation at first visit by Australian-born women and women of refugee background over baseline (B) and intervention (I) time periods for Hospital Networks X and Y**

|  | **Baseline 1**  **Jan-June 2014** | **Baseline 2**  **July-Dec**  **2014** | **Intervention 1**  **Jan-June**  **2015** | **Intervention 2**  **July –Dec 2015** | **Intervention 3**  **Jan- June**  **2016** | **Intervention 4**  **July- Dec**  **2016** |
| --- | --- | --- | --- | --- | --- | --- |
|  | **n (%)** | **n (%)** | **n (%)** | **n (%)** | **n (%)** | **n (%)** |
| **Hospital Network X** | | | | | | |
| **Attended 7 or more visits** | | | | | | |
| Australian-born women | 971 (58.8) | 1272 (74.5) | 1241 (74.9) | 1426 (80.2) | 1375 (80.7) | 1460 (81.9) |
| Women of refugee background | 246 (61.2) | 314 (77.0) | 336 (73.4) | 356 (78.6) | 407 (79.2) | 422 (83.6) |
| **Gestation 1^st^ visit <16 weeks*** | | | | | | |
| Australian-born women | 1125 (68.4) | 1068 (62.9) | 1093 (66.6) | 1098 (62.2) | 1117 (65.9) | 1141 (64.2) |
| Women of refugee background | 268 (67.0) | 237 (58.1) | 273 (59.7) | 242 (53.4) | 328 (63.8) | 286 (56.9) |
|  |  |  |  |  |  |  |
| **Hospital Network Y** | | | | | | |
| **Attended 7 or more visits**** | | | | | | |
| Australian-born women | 0 (0) | 512 (64.7) | 542 (61.7) | 733 (67.2) | 731 (69.7) | 779 (69.8) |
| Women of refugee background | 0 (0) | 101 (62.7) | 111 (62.0) | 169 (71.6) | 166 (60.8) | 160 (72.4) |
| **Gestation 1^st^ visit <16 weeks***** | | | | | | |
| Australian-born women | 539 (54.7) | 476 (45.8) | 493 (49.7) | 537 (50.9) | 460 (44.7) | 459 (42.0) |
| Women of refugee background | 118 (48.0) | 73 (32.6) | 78 (36.6) | 76 (32.3) | 90 (33.3) | 73 (33.8) |

* missing data for both Australian-born women and women of refugee background < 1.0% at each time point

** number of visits recoded from mid-2014, with missing data at B2=25% and I1=14%

*** missing data for Australian-born women <3.5% across time points; women of refugee background <1.0% at B1-I3, <2.5% at I4
