# Supplementary material for: Evaluation of systems reform in public hospitals, Victoria, Australia, to improve access to antenatal care for women of refugee background: An interrupted time series design
Source: PLoS Med. 2020 Jul 10;17(7):e1003089. doi: 10.1371/journal.pmed.1003089 (PMC7351141; doi:10.1371/journal.pmed.1003089)
Supplement: Attachment — Submitted filename: Bridging the Gap_PLOS_Reviewer Report.docx [file pmed.1003089.s006.docx]

Reviewer Report

Manuscript Title: ‘Bridging the Gap’: Systems reform in Victorian public hospitals to improve access to

antenatal care for women of refugee background evaluated using an interrupted time

series design

The manuscript is an insightful study based on two sets of expectant women, one - who sought refuge in Australia compared to two - native Australian women employing an interrupted time series analysis. The study is detailed and systematic with regard to the reported findings and results. The author(s) have employed a multivariate logistic regression model to determine the objectives of the study as well. They have mindfully reported the weaknesses of the study, especially with the limited time points employed in the time series. The findings procured with the multivariate logistic regression model spotlights answers to the research questions that the study attempts to investigate.

The following points incorporated in the forthcoming draft would further enrich the contents of the manuscript enabling the reader with a better perspective of the study:

1. The manuscript has concise information about the ‘Bridging the Gap’ (BG) initiative which is the principal intervention of the study.
   a. Creating a new Background Information section that includes the aims, objectives, purpose, target metrics and milestones, vision and goals of the BG initiative would help the reader draw sufficient information about the BG initiative. This step is important because the BG initiative needs to be described adequately as the context of the paper revolves around it.
2. The draft makes a mention of the hospitals that the two sets of expectant mothers visited for the study; however, it does not mention the inclusion and exclusion criteria of the selected hospitals.
   a. Adding the inclusion and exclusion criteria of the hospitals in the Methods section is essential to further inform the reader about the demographics of the sample population of expectant mothers who visited the hospitals leveraged for the study.

Overall, I would recommend acceptance of the manuscript with incorporations of the above two points.

Sincerely,

Dr. Shenoy

Dr. Amrita G. Shenoy, PhD, MBA, MHA, MSc

Assistant Professor and Graduate Program Director

Health Systems Management Program

School of Health and Human Services

College of Public Affairs

University of Baltimore

1420 N. Charles Street, Baltimore, Maryland, 21201 USA
