# Supplementary material for: Evaluation of systems reform in public hospitals, Victoria, Australia, to improve access to antenatal care for women of refugee background: An interrupted time series design
Source: PLoS Med. 2020 Jul 10;17(7):e1003089. doi: 10.1371/journal.pmed.1003089 (PMC7351141; doi:10.1371/journal.pmed.1003089)
Supplement: Attachment — Submitted filename: Response to reviews 8th February 2020.docx [file pmed.1003089.s007.docx]

5^th^ February 2020

Dear Editors

Re: Manuscript "‘Bridging the Gap’: Systems reform in Victorian public hospitals to improve access to antenatal care for women of refugee background evaluated using an interrupted time series design" (PMEDICINE-D-19-03494)

We thank you and the reviewers for their most helpful comments. Our response to these comments and suggestions are itemised below and highlighted using track-changes in the marked up manuscript.

We have checked and uploaded the two figures as TIF files with 300dpi resolution using the PACE tool.

With our thanks,

Jane Yelland and co-authors

| **Comments** | **Author Response** |
| --- | --- |
| **Editors** |  |
| 1. Title: Please revise to adhere to PLOS Medicine style, include a colon and a study descriptor in the second half the title. For example “Evaluation of systems reform in Victorian public hospitals to improve access to antenatal care for women of refugee background: an interrupted time series design” | Thank you. The title has been amended as suggested and with the removal of ‘Bridging the Gap’ to avoid two colons in the title. |
| 2. Abstract: please organise the abstract into “Introduction”, “Methods and Findings” followed by “Conclusions”. The last sentence of the methods and findings section should be a limitation of your methodology | The abstract has been organised into the three sub-headings. We have noted the following limitation in the last sentence to the methods and findings section:  *Using an interrupted time-series of observational data over the period of improvement is limited compared to using a randomization design which was not feasible in this setting.* |
| 3. Abstract: When reporting quantitative results, please be specific instead of using “about” or “around”   Abstract: please include participant demographics | We have removed these terms and included in the Abstract key participant demographics - maternal country of birth, interpreter requirements and parity, as follows:  *Refugee women were born in over 35 countries, and at one participating hospital network 40% required an interpreter. Compared to Australian-born women, women of refugee background were more likely to be having their second or subsequent baby, and have four or more children.* |
| 4. Data availability statement- authors cannot be responsible for requests for data access and should be a third party such as an ethics committee | Thank you. We have amended the data availability statement in the online platform noting that requests be made to the Royal Children’s Hospital HREC who have approved the study. |
| 5. At this stage, we ask that you include a short, non-technical Author Summary of your research to make findings accessible to a wide audience that includes both scientists and non-scientists. The Author Summary should immediately follow the Abstract in your revised manuscript. This text is subject to editorial change and should be distinct from the scientific abstract. | We have included the following Author Summary in the manuscript to be found on page 4. |
| 6. Please remove page numbers from the RECORD checklist as these are likely to change. Instead please use paragraphs and sections | This has been amended referencing paragraphs and sections. |
| 7. Did your study have a prospective protocol or analysis plan? Please state this (either way) early in the Methods section.   a) If a prospective analysis plan (from your funding proposal, IRB or other ethics committee submission, study protocol, or other planning document written before analyzing the data) was used in designing the study, please include the relevant prospectively written document with your revised manuscript as a Supporting Information file to be published alongside your study, and cite it in the Methods section. A legend for this file should be included at the end of your manuscript.  b) If no such document exists, please make sure that the Methods section transparently describes when analyses were planned, and when/why any data-driven changes to analyses took place.  c) In either case, changes in the analysis—including those made in response to peer review comments—should be identified as such in the Methods section of the paper, with rationale. | Yes, the study had a prospective study protocol, published as Open Access in Implementation Science in 2015, and this has been referenced in the paper.  The differences in the outcome specified in the protocol and presented in the paper (outlined the analysis section on page 15) and noted below.   - The primary outcome reported in this paper is focussed on access to antenatal care - Examining gestation of pregnancy at first hospital visit as a primary outcome(less than 16 weeks gestation used in the paper & less than 14 weeks gestation as specified in Table 3 of the protocol).   Australian clinical practice guidelines published in 2017/8 (reference 3) indicated that 16 weeks was well past the recommended gestation of the first antenatal visit.   - The analyses presented accounted for the clustering effects of women’s country of birth whereas in the protocol we only specified mothers within hospitals. Over the course of the research study, recording and access to maternal country of birth in the routine data system enabled this classification to be included within the study data. Accounting for country of birth within hospital system enabled a more robust adjustment for clustering (accounting for the likelihood of more similar outcomes within these groups of women) than had been previously envisaged using hospital data. |
| **Reviewer #1** |  |
| 1. I confine my remarks to statistical aspects of this paper. The general approach is fine but I have one suggestion and a few concerns that need to be addressed before I can recommend publication.   The authors use logistic regression. This isn't exactly wrong, but, by dichotomizing the outcomes they lose power and lose some ability to answer interesting questions. I suggest using some sort of count regression (e.g. negative binomial) model for the first aim and Cox models for the second aim. | Thank you for these suggestions.  The study was designed with the intent to evaluate whether women had attended 7 or more antenatal visits and conducted over a period where sufficient births were expected to provide adequate power for this comparison (as described in the protocol). This specific outcome was chosen because this indicates whether or not women have had the number of antenatal visits that would be recommended as a minimum. We agree that examining number of visits is of interest and these data are explored in Supplementary Table S2. While of interest we were cautious in the interpretation of the observed number of visits because frequent visits can reflect that the pregnancy was complicated and the mother required more care rather than quality of provision or engagement per se. Without detailed information regarding the complexity of each pregnancy we have been cautious to avoid over-interpretation of the number of visits.  Similarly for the second aim to evaluate whether care is begun sufficiently early in gestation we have used the dichotomisation which is most relevant for practice rather than considering Cox (time-to-event) models. Reasons for seeking early care can be varied and without this knowledge it was preferable to consider the dichotomisation of recommended initiation of antenatal care.  In practice these dichotomies in outcome may be readily monitored and are applicable for evaluation of services and quality improvement processes so their inclusion in this research helps increase applicability and translation to practice. |
| 2. In addition, the authors need to assess the assumptions of ITS analysis. In particular 1) Linear trend prior to intervention. Unless I am missing something, there are only 2 time points before the intervention, so this can't be assessed, but the assumption has to be mentioned. 2) That the characteristics of the population do not change over the course of the study. | Thank you. In contrast to ITS studies where data are sampled at specific time points, these data were routinely recorded continuously and the time points are derived by categorisation of the time. The analytic decision was made to use larger time spans for each period rather than increase the number of periods. This decision was primarily to maximise the number of women in each time period so that estimates could be made more precisely and comparisons between Australian born women and women of Refugee background were clearer (please see Figure 1 and Figure 2). We appreciate your thoughts with regard to the time points and have run the analysis again using three monthly intervals with no change in the findings (please see the table with the additional time points included at the end of our response) This is noted in the analysis section and discussion on page 27.  Thank you for your comment regarding the characteristics of the populations and change over time. A key indicator of changes in this population of women is their country of birth. Country of birth was examined over the time period of the study and little change was seen (please see Supplementary table S1 and page 18). |
| 3. Table 3 - I don't think age should be categorized; certainly not this way. Maybe use some sort of density plot, or maybe combine the youngest and oldest into a "high risk" category Also it isn't clear what the p value here is testing. Chi-square? Some sort of trend measure? | The categorisation helps with accessibility of the findings and allows for non-linearity in either risk or access according to age. As noted the youngest and oldest women would often experience the highest risks in epidemiological studies but this may be due to very different social, biological and behavioural profiles so it would be a preference to keep these groups distinct.  The p value is from a chi-squared test comparing the characteristics of Australian-born women and women of refugee background within each hospital network. This has been clarified in an additional footnote to the table. |
| 4. Figures - making the y axis go from 0 to 100 does show the full possible range, but it obscures the trends. Consider using 50 to 100. | We chose to show the range from 0 to 100 to visually illustrate that rates are generally high and felt this didn’t obscure the trends observed. In Figure 2 the lowest confidence interval almost approaches 30 and we preferred to keep the scale constant across the presentation. |
| **Reviewer#2** |  |
| 1. The manuscript is an insightful study based on two sets of expectant women, one - who sought refuge in Australia compared to two - native Australian women employing an interrupted time series analysis. The study is detailed and systematic with regard to the reported findings and results. The author(s) have employed a multivariate logistic regression model to determine the objectives of the study as well. They have mindfully reported the weaknesses of the study, especially with the limited time points employed in the time series. The findings procured with the multivariate logistic regression model spotlights answers to the research questions that the study attempts to investigate. | Thank you. |
| 2. The manuscript has concise information about the 'Bridging the Gap' (BG) initiative which is the principal intervention of the study.   Creating a new Background Information section that includes the aims, objectives, purpose, target metrics and milestones, vision and goals of the BG initiative would help the reader draw sufficient information about the BG initiative. This step is important because the BG initiative needs to be described adequately as the context of the paper revolves around it. | Thank you. We have included the aims of Bridging the Gap in the Methods section on page 7 which follows the description of the multiple initiatives, iteratively developed and implemented under the *Bridging the Gap* program. These are described in more detail in Table 1 including reference to publications reporting several of the initiatives. |
| 3. The draft makes a mention of the hospitals that the two sets of expectant mothers visited for the study; however, it does not mention the inclusion and exclusion criteria of the selected hospitals.   Adding the inclusion and exclusion criteria of the hospitals in the Methods section is essential to further inform the reader about the demographics of the sample population of expectant mothers who visited the hospitals leveraged for the study. | We have amended the Methods section and included additional contextual information on page 7 describing Australia’s universal health system, pathways into public maternity care and the selection of the hospital networks into *Bridging the Gap*. |
| **Reviewer #3** |  |
| 1. This is a fascinating study where the authors analyze the population of women receiving antenatal care at two hospital networks and try to assess differences based on refugee/immigration status. It would be interesting to try to tie this information to outcomes such as stillbirths, premature deliveries, obstetrical complications, maternal deaths. Could the authors do this? Could the authors comment on the link between antenatal care and outcomes in general? | Thank you.  The aim of the *Bridging the Gap* program was to implement and evaluate co-designed intervention strategies in maternity hospitals to improve access to healthcare for families of refugee background. The focus of the paper is on the primary outcome of access to antenatal care as an indicator of system improvement. While we appreciate the interest in the link between antenatal care and perinatal outcome this was not within the scope of the paper. |
| 2. The authors note that there is a higher incidence of poor birth outcomes amongst refugees yet by their data, there is a similar rate of antenatal visits of refugee women and Australian born women. Could the authors comment about this? | A number of factors are associated with poor birth outcomes including the number and timing of antenatal visits. We note on page 28 the strengths of the study and implications for policy, practice and research, and recognise the inherent limitation of using the number of antenatal visits as primary outcome measure for assessing the impact of the program initiatives. This is just one arm of the evaluation, with future papers reporting qualitative insights into system change. |

Table 4 using 3-monthly data (changes to original table highlighted):

**Table 4. Regression analyses for Hospital Networks X and Y - seven or more visits and gestation at first visit < 16 weeks**

| **Seven or more antenatal visits** | | | | | | |
| --- | --- | --- | --- | --- | --- | --- |
|  | **Hospital Network X*** | | | **Hospital Network Y**** | | |
|  | **Adj OR †** | **CI** | **P** | **Adj OR ‡** | **CI** | **P** |
| **Intervention**  **Baseline 1 & 2**  **Intervention 1-4** | 1.0 ref  1.06 | 0.95, 1.19 | 0.284 | 1.0 ref  0.88 | 0.81, 0.97 | 0.007 |
| **Time**  **(per 3 month period)** | 1.11 | 1.06, 1.16 | <0.001 | 1.06 | 1.04, 1.07 | <0.001 |
| **Country of birth**  **Australian-born**  **Refugee background** | 1.0 ref  1.07 | 0.46, 2.47 | 0.892 | 1.0 ref  0.96 | 0.72, 1.28 | 0.788 |
| **Parity**  **Nulliparous**  **Multiparous** | 1.0 ref  0.85 | 0.80, 0.89 | <0.001 | 1.0 ref  0.97 | 0.93, 1.02 | 0.262 |
| **Gestational Diabetes**  **No**  **Yes** | 1.0 ref  1.03 | 0.93, 1.13 | 0.631 | 1.0 ref  1.67 | 1.56, 1.78 | <0.001 |
| **Gestation at first visit less than 16 weeks** | | | | | | |
|  | **Hospital Network X***** | | | **Hospital Network Y****** | | |
|  | **Adj OR †** | **CI** | **P** | **Adj OR ‡** | **CI** | **P** |
| **Intervention**  **Baseline 1 & 2**  **Intervention 1-4** | 1.0 ref  1.06 | 0.80, 1.41 | 0.680 | 1.0 ref  1.21 | 1.07, 1.38 | 0.003 |
| **Time**  **(per 3 month period)** | 0.98 | 0.96, 1.01 | 0.185 | 0.94 | 0.93, 0.95 | <0.001 |
| **Country of birth**  **Australian-born**  **Refugee background** | 1.0 ref  0.88 | 0.31, 2.47 | 0.804 | 1.0 ref  0.60 | 0.44, 0.82 | 0.001 |
| **Parity**  **Nulliparous**  **Multiparous** | 1.0 ref  0.98 | 0.92, 1.05 | 0.627 | 1.0 ref  1.09 | 1.06, 1.12 | <0.001 |

**†** Analyses used were logistic regression adjusted for clustering by hospital and maternal country of birth

**‡** Analyses used were logistic regression adjusted for clustering by maternal country of birth

* No differential effect of intervention (p=0.189), time (p=0.772), parity (0.835) or diabetes (p=0.118) according to whether women were Australian-born or of a refugee background and attended seven or more antenatal visits at Hospital Network X.

** No differential effect of intervention (p=0.855), time (p=0.777), parity (0.140) or diabetes (p=0.404) according to whether women were Australian-born or of a refugee background and attended seven or more antenatal visits at Hospital Network Y.

*** No differential effect of intervention (p=0.456), time (p=0.570) or parity (0.816) according to whether women were Australian-born or of a refugee background and had a gestation of less than 16 weeks at first antenatal visit at Hospital Network X.

**** No differential effect of intervention (p=0.140), time (p=0.176) or parity (0.851) according to whether women were Australian-born or of a refugee background and had a gestation of less than 16 weeks at first antenatal visit at Hospital Network Y.
