# Supplementary material for: Evaluation of systems reform in public hospitals, Victoria, Australia, to improve access to antenatal care for women of refugee background: An interrupted time series design
Source: PLoS Med. 2020 Jul 10;17(7):e1003089. doi: 10.1371/journal.pmed.1003089 (PMC7351141; doi:10.1371/journal.pmed.1003089)
Supplement: Attachment — Submitted filename: Response to revision 20 Feb 2020 2.docx [file pmed.1003089.s008.docx]

26^th^ February 2020

Thank you to the PLOS Medicine team for comments and requested revisions. Please see our response as itemised below

|  | **Author Response** |
| --- | --- |
| **Request from the Editors** |  |
| 1. Title – can Australia be worked in please? | Australia has been included in the title. |
| 2. Abstract - I think some more summary demographic information in the abstract may be useful including mean age and add months to the dates. Please add not sufficiently powered to limitations? (as in line 207) | We have added months to the study dates and noted that that refugee women and Australian-born women did not differ by age at the time of birth.  The section referring to target sample size identifies that our original estimate of the proportion of women of refugee background giving birth at the participating hospitals (5%) was an underestimate. The study identified that of all women giving birth 10% were of refugee background. |
| 3. Author summary -please include bullet points | Bullet points have been included. |
| 4. Author summary- lines 41 and 42 perhaps not needed here | This bullet point has been removed. |
| 5. Please provide hospital names participating in Victoria so the study findings are more relevant. Please consider including a map of Victoria indicating the spread of sites participating, city names in Victoria if hospital names could be potentially identifying. | The hospital names have now been included in the manuscript on pages 6 & 7 |
| 6. Lines 44,45 we suggest you revise to “we co-designed and implemented multiple quality improvement and demonstration initiatives in universal health services, including four maternity hospitals” | Thank you. This has been revised (now lines 45,46) |
| 7. Lines 48-49- perhaps not relevant here? Suggest removing and incorporating in a previous point | Our preference is to leave this point as it highlights a unique approach to identifying women of refugee background (detailed in the paper) and the size of the population of interest. |
| 8. Line 58-60 perhaps not relevant here and we suggest removing | We have removed this bullet point. |
| 9. Is this a significant limitation that could cause overestimation? Please discuss in the conclusions as required : Country of birth was considered the best available proxy measure for identifying women of refugee background in routinely collected hospital data systems | Thank you. We have opted not to include a discussion in the conclusion given that the potential overestimation and underestimation is unknown.  We have detailed in the Methods section the rationale for why we consider that maternal country of birth is the best proxy to identify women of refugee background. While the method may cause an overestimation of women from certain countries, there are also numbers of women of refugee background who were born in countries that are not considered to be refugee source countries e.g. women whose families had fled countries of persecution and lived as refugees in neighbouring countries such as Pakistan, Kenya and Thailand. |
| 10. References- when citing multiple sources, please use single brackets for example Line 356 [27,28] | This has been corrected. |
| 11. Data – "some restrictions", please indicate what these restrictions (ethical, consent related etc) are and if data might be available on request. Please note that authors cannot be data contacts. | This has been amended in the online platform. |
| **Reviewer#1** |  |
| 1. The authors have responded to most of my issues satisfactorily. One remain issue regarding table 3, I wrote, asking them not to categorize age. They responded:   *The categorisation helps with accessibility of the findings and allows for non-linearity in either risk or access according to age. As noted the youngest and oldest women would often experience the highest risks in epidemiological studies but this may be due to very different social, biological and behavioural profiles so it would be a preference to keep these groups distinct.*  This is incorrect. Categorization often *obscures* nonlinearities, rather than illuminating them. I show some of this in my blog post https://medium.com/@peterflom/what-happens-when-we-categorize-an-independent-variable-in-regression-77d4c5862b6c | Thank you. We have responded to Dr Flom in a letter loaded on the online system as a separate file. |
| **Production** |  |
| FIGURES and GRAPHICS:  1. Please remove embedded figures from your manuscript file and ensure the most up to date versions of your figures are uploaded to our submission system as separate TIF or EPS files. Please do not remove the figure legends and captions.  2. Please ensure that all main figure files are cited in ascending numerical order in the main text of the article.  3. Please ensure that all main figures are referenced in the manuscript as Fig 1, Fig 2, etc. (including capitalization), rather than Figure 1, Figure 2, etc. Please note, however, that the file names themselves must not include the space, i.e. Fig1.tif, Fig2.tif, etc.  4. Please ensure that all main figure legends are placed after the paragraph containing their first in-text citation. | 1. The figure have been removed from the manuscript and we can confirm that the most up-to-date versions of the two figures are uploaded. Titles and figure legends have been retained in the manuscript. 2. All main figure files are cited in ascending numerical order. 3. Both figures are now referenced as Fig 1, Fig 2. 4. Figure legends are placed after the paragraph containing the first in text citation. |
| TABLES  1. Tables 1 and 4 use returns to control text wrapping. This is indicated by a [¶ or ↵] symbol when "Show Paragraph Marks" is turned on in Word. Please either split those cells into separate cells to achieve alignment or remove the returns. | Returns have been removed in Table 1 and Table 4 is split into separate cells. |
| METADATA  1. We still require confirmation of authorship and a declaration of any potential competing interests in relation to this manuscript from co-authors Wendy Dawson, Sue Casey and Mary Anne Biro. We have re-sent our authorship email, but also request that you contact these co-authors directly to ensure they respond.  2. Please provide a detailed Financial Disclosure statement on the submission form in full sentences, and include URLs, the initials of the receiving author, and specific grant numbers wherever possible.  3. Please ensure that the funders and grant numbers match between the Financial Disclosure field and the Funding Information tab in your submission form. Note that the funders must be provided in the same order in both places as well.  4. In accordance with PLOS' data policy, please ensure that your Data Availability Statement in the submission form clearly identifies how readers can access your data. Data cannot be available on request, or only accessible by contacting one of the authors. Note that your Data Availability Statement will be typeset as it is written, so please ensure it is in complete sentences and appears as you would like it to in the published version. | 1. All authors have been contacted and the confirmation and declarations have been completed. 2. The Financial Disclosure statement has been updated. 3. These have now been matched. We have now acknowledged in-kind contributions the support of the Victorian Government Infrastructure Fund (acknowledging the contribution supporting infrastructure on the institute to make this and other research possible). 4. This has been amended |
| ARTICLE FILE:  1. Please indicate the corresponding author in the byline by placing an asterisk (*) after their affiliation number.  2. Please include the corresponding author's email address on the title page of your manuscript, indicated by an asterisk (*). Only an asterisk and the email address itself are required.  3. Please ensure that all authors have at least one affiliation listed on the title page, and that all listed affiliations are attributed to at least one author. Affiliations should be listed in ascending numerical order in both the affiliation list and in the author byline.  4. The name for your co-author Christine East does not match across the article file and the submission form. Please ensure that all author names match verbatim, including the use of a middle name/initials and any special characters (accents, umlauts, etc.), as correct spelling and formatting of author names is vital for accurate indexing in PubMed.  5. Please ensure that your Ethics statement is available in the Methods section of your manuscript in its entirety. | 1. This has been amended. 2. Email included. 3. Please clarify how to access individual author affiliations in the online platform in order to amend this. Thank you. 4. Christine East’s name is now correctly referred to in the article file. 5. This has been checked. |
| REFERENCES:  1. Please ensure your references are in the style of PLOS Medicine (ICMJE/Vancouver style). The reference style guide can be found here: http://journals.plos.org/plosmedicine/s/submission-guidelines#loc-references. You can also find a downloadable EndNote file in these guidelines to assist you. Please do a manual check as well, as the EndNote file does not catch everything. Please note that the references will be typeset as they appear so please ensure the format is consistent.  2. Please put all reference citations in square brackets separated by either commas or dashes with no spaces, e.g. [1,2,3] or [1-3].  3. Please make sure all references are cited in ascending numerical order in the text. | 1. All references have been checked and amendments appear as tracked changes 2. References now correctly cited 3. Ascending order is confirmed. |
| SUPPORTING INFORMATION:  1. Please ensure that all Supporting Information files use the following format verbatim throughout the manuscript (including legends and all in-text citations): S1 Fig, S1 Table, S1 Text, S1 Data, etc. Each file type should start at 1 and increase numerically. Please note, however, that the file names themselves must use an underscore rather than a space, i.e. S1_Fig.tif, S1_Table.xlsx, S1_Text.doc, S1_Data.xlsx, S1_PRISMA_Checklist.docx, etc.  2. Please ensure that each supporting information file has a legend at the end of the manuscript file, after the Reference list.  3. Please note that your submission cannot contain any references to generic "supporting information" files and instead must refer to specific files using our naming convention (S1 Fig, S1 Table, S1 Text, S1 Data, SI PRISMA Checklist, etc.) | 1. Supporting information files have been amended with correct labels. 2. The legend has been included. 3. Thank you. This has been amended. |
