# Supplementary material for: Evaluation of systems reform in public hospitals, Victoria, Australia, to improve access to antenatal care for women of refugee background: An interrupted time series design
Source: PLoS Med. 2020 Jul 10;17(7):e1003089. doi: 10.1371/journal.pmed.1003089 (PMC7351141; doi:10.1371/journal.pmed.1003089)
Supplement: Attachment — Submitted filename: Response_3rd April 2020.docx [file pmed.1003089.s009.docx]

3^rd^ April 2020

Thank you again to the PLOS Medicine team for requested revision.

| **Request from the Editor** | **Author Response** |
| --- | --- |
| We ask that you present age as a continuous variable | We have presented age as a continuous variable with mean, standard deviation and p values. This is reported in Table 3 and with minor amendments to the text. |
