# Supplementary material for: Evaluation of systems reform in public hospitals, Victoria, Australia, to improve access to antenatal care for women of refugee background: An interrupted time series design
Source: PLoS Med. 2020 Jul 10;17(7):e1003089. doi: 10.1371/journal.pmed.1003089 (PMC7351141; doi:10.1371/journal.pmed.1003089)
Supplement: Attachment — Submitted filename: Response to production issues.docx [file pmed.1003089.s010.docx]

PLOS Medicine

3^rd^ June 2020

Dear Production Team

Re: Evaluation of systems reform in public hospitals, Victoria, Australia, to improve access to antenatal care for women of refugee background: an interrupted time series design

Thank you for your requested amendments to our manuscript. Please see our response to each request. The marked-up (current amendments added to last amendment requested in May) and ‘clean copy’ manuscript have been uploaded in the online system.

Kind regards, Jane Yelland

| FIGURES and GRAPHICS:  1. Please remove embedded figures from your manuscript file and ensure the most up to date versions of your figures are uploaded to our submission system as separate TIF or EPS files. Please do not remove the figure legends and captions. | The embedded figure (legends) have been removed from the manuscript and legends are now in Word following the captions.  The legends remain in the figures in the TIF files |
| --- | --- |
| TABLES:  1. Table 1 uses returns to control text wrapping. This is indicated by a [¶ or ↵] symbol when "Show Paragraph Marks" is turned on in Word. Please either split those cells into separate cells to achieve alignment or remove the returns. | Apologies for missing the one remain return. This has now been removed in Table 1. |
| ARTICLE FILE:  1. Please ensure that all authors have at least one affiliation listed on the title page, and that all listed affiliations are attributed to at least one author. Affiliations should be listed in ascending numerical order in both the affiliation list and in the author byline. | Affiliations are now listed in ascending numerical order. I have include departments where appropriate. |
| REFERENCES:   1. Please put all reference citations in square brackets separated by either commas or dashes with no spaces, e.g. [1,2,3] or [1-3].   2. Please make sure all references are cited in ascending numerical order in the text. | All reference citations are in square brackets. References are cited in ascending numerical order. Some of these citations are in Table 1 and have been ordered according to the placement of Table 1 in the manuscript. |
| TITLE  Thank you to the team for responding to my email about an error that had crept in to the tile and suggested grammatical correction. This has been amended on the manuscript and in the submission system. | Amended title:  Evaluation of systems reform in public hospitals, Victoria, Australia, to improve access to antenatal care for women of refugee background: an interrupted time series design |
